# Unmasking the Unexpected: Renal Arteriovenous Malformation in a Young Puerto Rican Patient With Persistent Hypertension

**DOI:** 10.7759/cureus.114715

**Published:** 2026-08-18

**Authors:** Milton M Rivera-Valentin, Gretchen C Abarca-Riancho, Cristina M Perez, Brian Torres Mercado, Edwin O Molina Torres

**Affiliations:** 1 Internal Medicine, Hospital Damas, Ponce, PRI; 2 Cardiology, Mayagüez Medical Center, Mayagüez, PRI; 3 Cardiovascular Disease, Centro Médico Episcopal San Lucas, Ponce, PRI

**Keywords:** catheter angiography, coil embolization, endovascular embolization, renal angiography, renal arteriovenous malformation, renovascular hypertension, secondary hypertension, young adult

## Abstract

Renal arteriovenous malformations (AVMs) are uncommon vascular abnormalities that may present a diagnostic challenge, particularly in young patients undergoing evaluation for persistent hypertension. We present the case of a 20-year-old woman with hypertension that remained elevated despite treatment with lisinopril and extended-release nifedipine. During outpatient cardiology follow-up, her blood pressure was 163/91 mmHg, prompting further evaluation for a secondary cause. Renal ultrasonography demonstrated asymmetric kidney size, with the left kidney smaller than the right. Subsequent computed tomography angiography of the chest, abdomen, and pelvis demonstrated a normal aorta without evidence of coarctation and no significant stenosis of the main renal arteries. Despite unrevealing noninvasive vascular imaging, persistent clinical suspicion for an underlying renal vascular abnormality prompted selective renal angiography. Angiography revealed a hypervascular nidus in the lower pole of the left kidney supplied by a tortuous branch of the left main renal artery, consistent with a slow-flow renal AVM. Superselective embolization of the feeding vessel was performed through right distal radial access using a detachable microcoil. Final angiography demonstrated successful occlusion of the targeted AVM while preserving perfusion to the surrounding renal parenchyma. The procedure was completed without immediate complications. At four-month follow-up, hypertension persisted and required intensification of antihypertensive therapy, suggesting that the AVM could not be established as the sole cause of the patient's hypertension. This case highlights the importance of considering uncommon renal vascular abnormalities in young patients with severe hypertension and renal asymmetry when the initial evaluation is inconclusive. Selective renal angiography can provide detailed anatomic characterization while simultaneously offering an opportunity for targeted endovascular therapy.

## Introduction

Renal arteriovenous malformations (AVMs) are uncommon vascular abnormalities characterized by abnormal communications between the renal arterial and venous systems that bypass the normal capillary network. These lesions may be congenital or acquired and can exhibit variable vascular architecture and flow characteristics [1]. Renal AVMs may also be characterized by their angiographic architecture, including the number and configuration of feeding arteries, abnormal vascular communications, and draining veins, features that can influence the selection of an appropriate endovascular treatment strategy [2]. Although hematuria is the most frequently reported clinical manifestation, renal AVMs may also present with hypertension, flank pain, renal dysfunction, or, in larger shunts, hemodynamic consequences such as high-output heart failure [1,2].

The evaluation of hypertension in young adults warrants consideration of secondary causes, particularly when blood pressure remains elevated despite pharmacologic therapy or when clinical findings suggest an underlying renal or renovascular process. Renal ultrasonography and computed tomography angiography (CTA) are commonly used during the evaluation of renal and renovascular abnormalities; however, smaller or anatomically complex vascular lesions may not always be adequately characterized with noninvasive imaging. Selective renal angiography provides detailed visualization of vascular architecture and remains an important diagnostic modality when suspicion for a renal vascular abnormality persists [1,2].

Recognition of renal AVMs is clinically important because these vascular abnormalities may be associated with hypertension and other clinically significant manifestations. Furthermore, catheter angiography can transition directly from diagnosis to therapy via selective endovascular embolization, thereby targeting abnormal vascular communications while minimizing injury to unaffected renal parenchyma [2,3]. We present the case of a young woman with persistent hypertension and renal asymmetry in whom noninvasive evaluation did not identify a typical renovascular etiology. Selective renal angiography ultimately demonstrated a left renal AVM that was treated with superselective coil embolization. This case emphasizes the importance of maintaining clinical suspicion for uncommon renal vascular abnormalities when the initial evaluation for secondary hypertension is unrevealing, while recognizing that identification of an AVM does not necessarily establish it as the sole cause of hypertension.

## Case presentation

A 20-year-old woman was incidentally found to have markedly elevated blood pressure while using a publicly available automated blood pressure machine at a retail store, with a systolic blood pressure exceeding 200 mmHg and a diastolic blood pressure of approximately 90 mmHg. She subsequently sought medical evaluation and was diagnosed with hypertension. Despite initiation of antihypertensive therapy with lisinopril 15 mg and extended-release nifedipine 30 mg orally daily, her blood pressure remained persistently elevated, prompting further investigation for a secondary cause of hypertension.

As part of the evaluation for secondary hypertension, testing for primary hyperaldosteronism was unrevealing. Renal ultrasonography demonstrated asymmetric kidney size, with the left kidney smaller than the right. Given the patient's young age, persistent hypertension despite pharmacologic therapy, and renal asymmetry, further evaluation for a renovascular etiology was pursued. CTA of the chest, abdomen, and pelvis demonstrated a normal aorta without evidence of coarctation and no significant stenosis of the main renal arteries. Despite these findings, clinical suspicion for an underlying renal vascular abnormality remained.

The patient subsequently underwent selective renal angiography. Angiographic evaluation of the left renal artery demonstrated a hypervascular nidus involving the lower pole of the left kidney. The lesion was supplied by a tortuous branch arising from the left main renal artery and was consistent with a slow-flow renal AVM.

**Figure 1:**
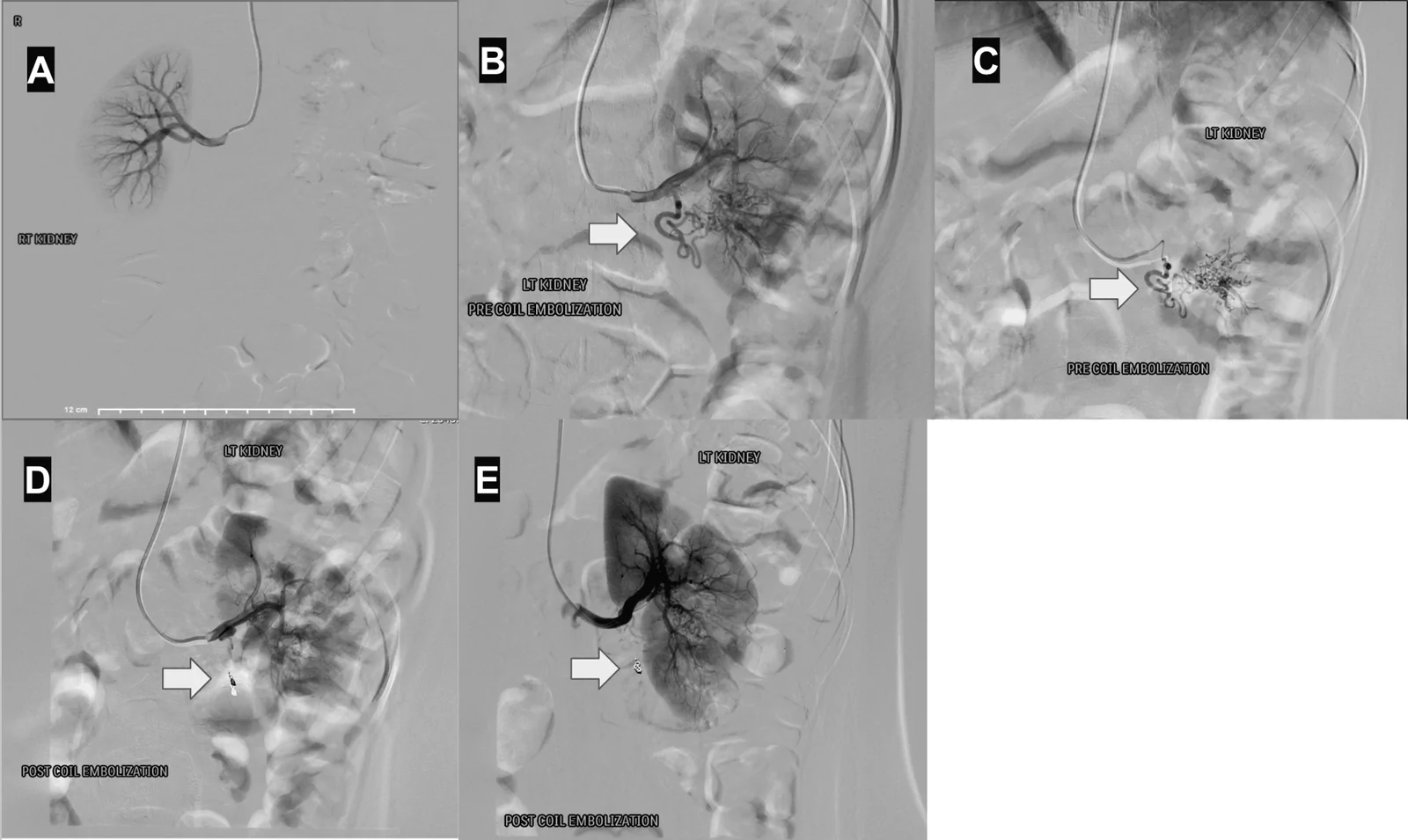
Selective renal angiography demonstrating a left renal arteriovenous malformation before and after coil embolization. (A) Selective right renal angiography demonstrating normal right renal arterial vasculature for comparison. (B-C) Pre-embolization selective left renal angiographic views demonstrating abnormal tortuous vascularity consistent with a renal AVM; the arrows indicate the abnormal vascular nidus corresponding to the AVM. (D-E) Post-embolization selective left renal angiographic views demonstrating angiographic occlusion of the targeted vascular malformation with preservation of perfusion to the remaining renal parenchyma; the arrows indicate the deployed embolization coil at the treated AVM site. AVM: arteriovenous malformation.

Given the angiographic findings and the presence of a discrete feeding vessel amenable to superselective catheterization, endovascular treatment of the lower-pole AVM was pursued. Right distal radial arterial access was obtained, and superselective catheterization of the feeding vessel was performed. The vessel was embolized with a 2-mm × 4-cm Interlock (Boston Scientific Corporation, Marlborough, MA, USA) detachable microcoil. Final angiography demonstrated no opacification of the targeted lower-pole AVM, with preservation of perfusion to the surrounding renal parenchyma. The angiographic findings before and after coil embolization are shown in Figure 1.

The procedure was completed without immediate complications. At four-month follow-up, her blood pressure remained elevated at 163/91 mmHg. Given persistent hypertension, lisinopril was increased to 20 mg orally daily, chlorthalidone 25 mg orally daily was initiated, and extended-release nifedipine 30 mg orally daily was continued.

## Discussion

Renal AVMs are rare vascular abnormalities characterized by abnormal communications between the renal arterial and venous systems. Their clinical presentation varies according to the size, location, angioarchitecture, and hemodynamic characteristics of the lesion. Hematuria is the most frequently reported manifestation; however, renal AVMs may also present with hypertension, flank pain, renal dysfunction, or, particularly with larger high-flow lesions, high-output heart failure [1,2]. The absence of hematuria in our patient is noteworthy because severe hypertension was the primary clinical manifestation that prompted investigation and ultimately led to identification of the AVM.

The relationship between renal arteriovenous abnormalities and hypertension is complex and incompletely understood. One proposed mechanism involves relative hypoperfusion of renal parenchyma distal to the vascular abnormality, resulting in increased renin secretion and activation of the renin-angiotensin-aldosterone system [3]. Altered intrarenal hemodynamics and vascular shunting may therefore contribute to persistent or severe hypertension even in the absence of conventional renal artery stenosis. Although renin-mediated hypertension has been described in patients with renal arteriovenous abnormalities, the contribution of renin-angiotensin-aldosterone system activation may vary among patients. This mechanism is particularly relevant when evaluating marked hypertension in a young patient, in whom secondary causes should be carefully considered. In our patient, evaluation for primary hyperaldosteronism was unrevealing, prompting continued investigation for alternative secondary causes of hypertension.

Importantly, the presence of the renal AVM in this patient does not establish it as the sole cause of her hypertension. The lesion was discovered during the evaluation of severe hypertension in the setting of renal asymmetry and an otherwise unrevealing noninvasive renovascular assessment. The decision to treat the AVM was based on its angiographic identification and on the anatomy being amenable to targeted endovascular therapy, rather than on the assumption that embolization alone would normalize the patient's blood pressure.

The diagnostic evaluation in this case illustrates an important limitation of relying exclusively on noninvasive vascular imaging. Renal ultrasonography demonstrated asymmetric renal size, with the left kidney smaller than the right, raising suspicion for an underlying renal or renovascular process. CTA subsequently excluded aortic coarctation and did not demonstrate significant stenosis of the main renal arteries. Nevertheless, these findings did not fully explain the patient's hypertension. Although ultrasonography, Doppler imaging, CTA, and magnetic resonance angiography can identify renal vascular abnormalities, digital subtraction angiography remains an important modality for defining the vascular anatomy of renal AVMs [1,2]. Selective angiography is particularly valuable because it can delineate feeding vessels, abnormal vascular communications, and venous drainage with sufficient detail to guide treatment.

In our patient, selective renal angiography revealed a slow-flow AVM involving the lower pole of the left kidney, supplied by a tortuous branch of the main renal artery. The lesion was amenable to superselective endovascular treatment. This approach reflects a central principle of endovascular management: treatment should target the abnormal vascular communication while preserving as much functional renal parenchyma as possible [2,4].

Historically, surgical approaches, including partial or total nephrectomy, were used for symptomatic renal AVMs. Advances in endovascular techniques have made transcatheter embolization an important kidney-preserving alternative [1,4]. Embolic options include metallic coils, liquid embolic agents, and other materials selected according to vascular anatomy and flow characteristics [2,5]. Selection of the embolic agent should be individualized according to the angioarchitecture and hemodynamic characteristics of the AVM. Liquid embolic agents may facilitate penetration of the nidus in lesions in which direct nidus occlusion is required, whereas coils may be selected when a discrete feeding vessel can be safely and superselectively targeted [2,5]. In the present case, the slow-flow lesion had a discrete feeding vessel that was amenable to superselective catheterization, and coil embolization resulted in angiographic occlusion of the targeted AVM while preserving perfusion to the surrounding renal parenchyma, without immediate procedural complications.

An important aspect of this case is the persistence of hypertension despite successful angiographic occlusion of the AVM. At four-month follow-up, the patient's blood pressure remained elevated at 163/91 mmHg and required intensification of antihypertensive therapy with an increase in lisinopril to 20 mg daily and the addition of chlorthalidone 25 mg daily, while extended-release nifedipine 30 mg daily was continued. Persistent hypertension despite technically successful embolization suggests that blood pressure regulation in this patient may be multifactorial and highlights that angiographic success does not necessarily result in immediate or complete resolution of hypertension. Accordingly, the association between the renal AVM and hypertension in this patient should be interpreted cautiously, as the persistence of hypertension after embolization does not establish the AVM as the sole cause. Continued blood pressure monitoring, optimization of antihypertensive therapy, and longitudinal clinical follow-up are therefore warranted.

This case emphasizes several clinically relevant lessons. First, severe hypertension in a young adult warrants a thorough investigation for secondary causes even when the patient is otherwise minimally symptomatic. Second, renal asymmetry may provide an important clue to an underlying renal or renovascular abnormality. Third, the absence of main renal artery stenosis on CTA does not exclude other potentially clinically significant renal vascular abnormalities. Finally, when clinical suspicion persists despite inconclusive noninvasive imaging, selective renal angiography can provide detailed anatomic characterization and, when appropriate, offer a route for targeted endovascular therapy. The persistence of hypertension after technically successful embolization further emphasizes that identification and treatment of a renal vascular abnormality should not replace continued evaluation and guideline-directed management of hypertension.

## Conclusions

Renal AVMs are uncommon renal vascular abnormalities that may be associated with hypertension and should be considered among potential renovascular etiologies in appropriately selected young patients. This case demonstrates that an unrevealing noninvasive vascular evaluation does not necessarily exclude a clinically significant renal vascular abnormality. Renal asymmetry and persistent severe hypertension may prompt consideration of less common renovascular etiologies when the initial diagnostic workup is inconclusive.

Selective renal angiography can provide detailed anatomic characterization and, when appropriate, enable simultaneous endovascular treatment. Selection of an embolization strategy should be individualized according to lesion angioarchitecture and flow characteristics, with the goal of treating the abnormal vascular communication while preserving functional renal parenchyma. The persistence of hypertension despite technically successful embolization in this patient indicates that the AVM cannot be established as the sole cause of her hypertension and underscores the importance of continued antihypertensive management and longitudinal follow-up.
